# The Mediating Role of Resilience and Life Satisfaction in the Relationship between Stress and Burnout in Medical Students during the COVID-19 Pandemic

**DOI:** 10.3390/ijerph19052822

**Published:** 2022-02-28

**Authors:** Ivone Duarte, Ana Alves, Ana Coelho, Ana Ferreira, Beatriz Cabral, Bebiana Silva, João Peralta, Juliana Silva, Pedro Domingues, Pedro Nunes, Carla Serrão, Cristina Santos

**Affiliations:** 1 Department of Community Medicine, Information and Decision in Health (MEDCIS), Faculty of Medicine, University of Porto, 4200-450 Porto, Portugal; csantos@med.up.pt; 2 CINTESIS-Center for Health Technology and Services Research, Faculty of Medicine, University of Porto, 4200-450 Porto, Portugal; 3 Faculty of Medicine, University of Porto, 4200-450 Porto, Portugal; up201604246@edu.med.up.pt (A.A.); up201606355@edu.med.up.pt (A.C.); up201508918@edu.med.up.pt (A.F.); up201304008@edu.med.up.pt (B.C.); up201307835@edu.med.up.pt (B.S.); up201604454@edu.med.up.pt (J.P.); up200801044@edu.med.up.pt (J.S.); up201606261@edu.med.up.pt (P.D.); up201504684@edu.med.up.pt (P.N.); 4 School of Education, Polytechnic of Porto, 4200-465 Porto, Portugal; carlaserrao@ese.ipp.pt; 5 INED-Centre for Research and Innovation in Education, School of Education, Polytechnic of Porto, 4200-465 Porto, Portugal

**Keywords:** COVID-19, SARS-CoV-2, burnout, stress, resilience, medical students

## Abstract

Following the WHO’s declaration of a public health emergency due to the COVID-19 outbreak, the subsequent quarantine and confinement measures that were adopted, including distance learning measures, were shown to have caused a significant deterioration in the mental health of medical students. The goal of this study was to explore the mediating role of resilience and life satisfaction in the relationship between perceived stress and burnout among medical students in the context of COVID-19. A transversal assessment was performed using an online questionnaire, to which 462 students responded. The instruments applied were the Perceived Stress Scale-10, the Resilience Scale-25 items, the Satisfaction with Life Scale (SWLS), and the Burnout Scale (Oldenburg Inventory). A regression model was estimated for each dimension of burnout. The results revealed that resilience and life satisfaction play a mediating role in the association between stress and the dimensions of burnout. This suggests that measures of promoting mental health based on resilience and improving perceptions of life should be implemented.

## 1. Introduction

COVID-19 was first identified in December 2019 in Wuhan, China [1]. On 30 January 2020, the WHO declared the outbreak of the novel coronavirus a public health emergency of international concern, the WHO’s highest level of alert [2]. During this unprecedented pandemic era, most countries adopted exceptional and temporary measures regarding the epidemiological situation of the COVID-19 disease. Among these measures, the suspension of in-person teaching activities particularly stands out, with those activities subsequently being offered at a distance through so-called emergency remote teaching [3,4]. Mandatory confinement, quarantine, isolation, and social distancing have already had negative effects on the mental health of the general population, where exponential growth in stress levels and symptoms of depression and anxiety has been observed [3]. Looking at the university context specifically, the study of Zis et al. of a sample of 154 first-through sixth-year medical students in Cyprus found a significant deterioration in their mental health from the pre- to a post-COVID-19 period (pre-COVID-19 58.8 ± 21.6 vs. COVID-19 48.3 ± 23, *p* < 0.001), related with the adoption of emergency remote teaching methods [5]. In the Australian context, in March 2020, Lyon et al. conducted a study to evaluate the impact of COVID-19 on the mental health of a sample of 297 medical students. The results indicate that more than two-thirds displayed a deterioration in their mental health [6].

Medical students can experience stress when curricular demands exceed their resources to manage them [7], and a higher prevalence of perceived stress compared to both the general population and students of other academic areas has been reported [8,9]. As a population being initiated into the healthcare profession, medical students are also at higher risk for depression, anxiety, and psychological distress than their non-medical peers, as well as a higher risk of burnout, emotional exhaustion, and increased levels of fatigue [10]. 

Furthermore, medical students have a lower likelihood of seeking help, possibly stemming from the stigma associated with this group in particular receiving mental health care [11]. In addition to the level of perceived stress, studies have reported psychosocial consequences, such as increased levels of anxiety and depression, as well as diminished quality of life and an increased prevalence of burnout [12,13]. The study by Saraswathi also demonstrated a significant increase in the prevalence of anxiety (33.2%) and stress (24.9%) during the COVID-19 pandemic [14].

If even under normal circumstances, it is highly likely that most medical students will be affected by burnout to some degree by their studies [15]; it is expected that the pandemic and the stress factors resulting from the uncertainty and abrupt changes would exacerbate this issue. Within non-medical university students, the impact of the COVID-19 crisis resulted in higher rates of stress and perceived social isolation [16] and a higher prevalence of anxiety and depression than before the COVID-19 pandemic [17]. Medical students, who experienced a deterioration of their mental health during the pandemic, have also expressed concerns about the impact of this situation on the functioning of their studies, since it compromises an adequate preparation for clinical practice [6]. The COVID-10 pandemic has affected the preclinical and clinical aspects of medical education, with an abrupt transition in the learning format to online and small group education [18]. Despite the opportunities associated with e-learning, it may not yet be adequately adapted to some disciplines requiring a hands-on approach, such as medicine [19]. During the era of the COVID-19 pandemic, many countries worldwide have adopted very strong measures. The social isolation, the uncertainty and fear, the changes arising from the teaching–learning process, namely emergency remote teaching [4], are only some of the factors associated with the pandemic that may contribute to increased exhaustion, fatigue, and stress in medical students.

Burnout is a term that was first used by Herbert Freudenberger, described as a mental disorder resulting from prolonged exposure to career-related stress factors; it was later defined as a multidimensional occupational syndrome (Burnout Syndrome) [20]. In 2019, the syndrome was included in the International Classification of Diseases as an occupational phenomenon [21]. Added to the study-related workload of medical students are the demands and responsibilities imposed upon them due to the nature of their future profession, one dedicated to providing care to humans and thus with a reduced tolerance for error, conditions that favor the development of stress and anxiety [22,23]. A global study, from 12 countries demonstrated alarmingly high rates of mental health problems, burnout, substance abuse, and mental stress in medical students [24]. 

Although there are studies on burnout in students, little importance has been given to the measure of the construct. In the vast majority of studies, academic exhaustion was measured by the Maslach Burnout Inventory-General Survey (MBI-GS) [25]. Criticism of this led several authors to propose alternative assessment tools [26,27]. Furthermore, some scholars have criticized the psychometric qualities of the MBI-GS [28,29]. For this reason, we decided to use the Oldenburg Burnout Inventory (OLBI) [26,30,31]. 

The Oldenburg Burnout Inventory was originally developed by Demerouti and Nachreiner, who suggested two burnout dimensions, disengagement, and exhaustion, applicable to professions outside human services occupations [32]. It has many versions across various occupational groups; in Portugal, there is a version for students. The exhaustion subscale is related to feelings of emptiness, excessive workload, need for rest, and physical, cognitive, and emotional exhaustion, and the disengagement subscale is related to negative and cynical behaviors and attitudes regarding work [33].

Burnout plays a significant role in the overall well-being of medical students and is associated with poor academic performance, sleep disturbance, risk of severe mental illness, and substance use disorder [34]. It is important to investigate the burnout phenomenon in university students because the research indicates that burnout begins in medical school and continues after graduation [35]. One factor that can explain why some physicians possess the capacity to cope effectively with stress and the ability to bounce back from stressors is resilience [36]. Keeton et al. [37] describe resilience as a protective factor against burnout and as a variable that cushions the impact of the negative effects of occupational stressors. West et al. [36] examined a sample of 5445 physicians and concluded that resilience was inversely associated with burnout symptoms. 

The literature defines psychological resilience as the capacity to maintain a state of equilibrium, regardless of the presence of extremely adverse circumstances [38,39,40]. Another definition points to the ability to respond to stress healthily, such that goals are achieved with a minimum of psychological and physical distress [41,42]. Therefore, resilient individuals are usually prepared to recover from challenges while simultaneously learning and gathering strength from the experience [42]. Similarly, “resilience” refers to the internal resources that the subject possesses to be able to deal with stress factors in a manner that leaves a minimal psychological impact, while simultaneously enjoying personal development and building new adaptive mental “tools” [40]. Simply put, resilience is described as an adaptive skill generated by the interaction between individuals and their respective environments [41]. It can be easily understood, then, that psychological resilience is malleable and can be practiced, having recently been identified as a priority in medical training [13,42,43].

To this end, one strategy that could facilitate the development of adaptation mechanisms among medical students might be promoting personal reflection on past experiences and the consequent recognition that not all life events can have positive outcomes [44]. Furthermore, articles suggest that students who report greater commitment to self-care activities have lower rates of perceived stress and greater quality of life; in other words, those with more balanced lifestyles demonstrate greater resilience [42]. In the same vein, good levels of social belonging, relationships, and support are characteristics that contribute to resilience [45,46].

On the one hand, some studies demonstrate an association between resilience in medical students and lower levels of psychological distress, greater life satisfaction, happiness, greater quality of life, fewer symptoms of anxiety, and a greater subjective perception of well-being [41]. On the other, some studies point to higher levels of perceived stress and lower resilience among medical students compared to the general population [42]. For example, a study that included Canadian students stratified by age and gender with controls from the general population found that the former have lower resilience scores than the latter [47]. Given this, research on resilience in medical students must continue to be developed so that it can be better characterized and subsequently improved. On another note, it is known that resilience is one factor that can reduce burnout. Therefore, greater resilience is associated with less burnout and greater tolerance for uncertainty [40]. Furthermore, one study found that students who are resilient against burnout, or who recovered from it, have lower levels of stress and fatigue in the first year of follow-up compared with students who are experiencing burnout [48]. One article looking at the correlation between academic burnout and the psychological well-being of medical students points to the premise that the greater the level of burnout, the harder it is for students to make a positive assessment of the present moment [49]. Another article notes the positive correlation between well-being and resilience among Chinese medical students. Moreover, the authors report that the stress factor negatively influenced students’ life satisfaction, leading to the development of depression [49]. An important personal characteristic that contributes to resilience is psychological flexibility, which is understood as the individual capacity to develop and maintain full awareness of one’s thoughts and feelings. Psychological flexibility is correlated with greater perceived quality of life and emotional well-being. The same study mentions that medical students who can manage their own emotions and thoughts in this way are capable of being more resilient and less prone to psychological distress and burnout [50].

Life satisfaction is a cognitive and global assessment of individuals’ quality of life and well-being [51]. Burnout and stress negatively impact the quality of life, leading to various negative outcomes. Moreover, previous studies have found that individual subjective well-being is an important variable in attenuating symptoms of stress and burnout [52]. Thus, subjective well-being is expected to act as a mediator in the relationship between stress and burnout. Regardless of the effects of COVID-19, several studies have already indicated the effect of the relationship between burnout, resilience, and psychological well-being [11,12,13]. However, to the best of the authors’ knowledge, no study has evaluated this relationship in medical students during the COVID-19 pandemic. Thus, this issue deserves particular attention in the context of a pandemic.

Here, we must highlight the necessary role of universities as entities of health promotion, given the symbiotic relationship between health and education. To that end, university institutions must develop strategies to promote mental health that consider students’ emotional and psychological needs, with the aim of capitalizing on efforts to minimize possible structural risk factors and optimize students’ adaptive mechanisms [50]. Therefore, practicing resilience and investing in well-being becomes increasingly relevant across the various domains of an individual’s life [52].

Keeping in mind the existing evidence that resilience appears to be a protective factor against burnout and positively related to life satisfaction, the goal of this study is to explore the mediating roles of resilience and life satisfaction in the relationship between perceived stress and burnout among medical students in the context of COVID-19.

## 2. Materials and Methods

### 2.1. Study Design and Participants

This study included a transversal assessment of students attending a medical school in northern Portugal. The data were collected through a survey shared through both social media and the school’s official communications channels. The data collection period was between 6 May 2020 and 29 May 2020. Responses were collected using the Google Forms platform. 

The ethical procedures were carried out in accordance with the Helsinki Declaration, whereby the Ethics Committee of the São João hospital complex analyzed and approved the study (ref. 98/2020). All participants gave their informed consent online, following the General Directives of the General Data Protection Regulations for clinical research [53]. 

In total, 462 students responded to the survey, with an average age of 22 (range = 18–42). Table 1 describes the characteristics of the sample.

### 2.2. Survey Questionnaire

The survey comprised 3 separate parts: collecting sociodemographic data, evaluating students’ resources, and evaluating their habits. The psychological variables analyzed were perceived stress, psychological resilience, life satisfaction, and burnout. 

The Perceived Stress Scale-10 (PSS-10) from Cohen and Williamson [40], adapted to Portugal by Trigo [54] with a good internal consistency (Cronbach’s Alpha: 0.874), is a global measure that evaluates to what extent life events are perceived as stress inducers. The studies performed on the Portuguese population concluded that the PSS-10 presents good psychometric qualities, with the Portuguese version shown to be generally robust, easy to understand, quick to fill out, and simple to score. The PSS-10 consists of 10 questions about the frequency (0–never; 1–almost never; 2–sometimes; 3–fairly often; 4–very often) with which a general life circumstance has occurred over the last month, without specifying any situation in particular. In the studies on the Portuguese population, an equivalence was established between the scores of the PSS-10 and the respective percentile; raw scores above the 80th percentile were considered indicators of pathology [54].

The Resilience Scale ([39]; adapted by Oliveira and Machado [55]) consists of 25 items organized according to a 7-point Likert scale (where 1 is “totally disagree”, 4 is “neither agree nor disagree”, and 7 is “totally agree”, referring to the respondent’s agreement with each statement); the final score can range from 25 to 175 points, with higher scores indicating higher resilience. A score below 121 is considered indicative of “reduced resilience”; a score between 121 and 145 is considered “moderate resilience”; and a score above 145 is considered “high moderate resilience” to “high resilience” [55]. Applied to a sample of higher education students, this scale demonstrated good internal consistency (Cronbach’s Alpha: 0.89). The scoring and interpreting of results were as follows: 25–120 low resilience, 121–145 moderate resilience, and 145–175 high resilience. 

The Satisfaction with Life Scale [56] aims to evaluate the cognitive component of subjective well-being and is composed of 5 items. Each item is an affirmation to which the respondent must attribute a level of agreement using a 7-point scale (from 1: “strongly disagree” to 7: “strongly agree”). The scale was adapted to the Portuguese population [57] with an α of 0.77. The result of the scale is determined by adding the scores of the five items, ranging from 5 to 25 points. Higher scores suggest greater life satisfaction. This scale is characterized by acceptable and high internal consistency (original version: α = 0.87 and the Portuguese version: α = 0.77). 

The Oldenburg Inventory (OLBI) [29] was adapted for Portuguese students by Campos et al. [33] and seeks to evaluate their level of burnout. The inventory consists of 15 items and includes two subscales: the exhaustion subscale and the disengagement subscale. In the Portuguese version [33], the internal consistency of the exhaustion subscale was α = 0.565 and the disengagement subscale was α = 0.700. Despite confirming the two-dimensional structure of the Portuguese version of OLBI-S, it had limitations regarding the convergent validity and internal consistency; however, concurrent and divergent validity were good [33].

### 2.3. Statistical Analysis

The data were analyzed using the SPSS^®^ Statistics software (version 26, IBM, Armonk, NY, USA). Separate multiple linear regressions were done for each outcome (disengagement burnout and exhaustion burnout). The regression assumptions were checked. To decide which variables would be included in each multiple linear regression, simple linear regressions were performed for each of the variables: gender, age, civil status (married or de facto union; divorced or single), has children, academic year, attending the academic institution for the 1st time, public institution of secondary education, special status, being away from home, living with how many other people (0; 1; 2; 3; 4 or more), leaves the house how often (occasionally; 1–2 times per week; more than 3 times per week), being involved in some kind of volunteering, possessing all means to keep up with classes, taking nutritional supplements or stimulants, getting regular physical exercise before the pandemic, extracurricular activities, stressful life event in the last year, psychological support in the last year, loss of a family member/friend, taking a COVID test (no, but would like to; no, doesn’t want to; yes, has the result). All the variables correlated with outcomes with *p* ≤ 0.20 in the simple linear regressions were included in the multiple regressions, in accordance with the methods described by Greenland. In multiple linear regressions, *p* ≤ 0.05 was considered significant [58].

## 3. Results

Regarding psychological resilience, 129 students (28%) demonstrated reduced resilience, 236 (51%) moderate resilience, and 97 (21%) high resilience. The students sampled scored an average resilience of 129, with a standard deviation of 20 and a range of 54–174. Regarding perceived stress, the students scored an average of 21, with a standard deviation of 7 and a range of 2–38. As for life satisfaction, the median score of the students sampled was 20, with a range of 5–25. Finally, regarding burnout, the students scored an average of 40, with a standard deviation of 7 and a range of 22–61. 

An extensive representation of the results obtained is shown in Table 2 and Table 3. The model used consists of four steps: in the first, all independent variables were adjusted through the multiple linear regression, and of these, those that were shown to be associated with exhaustion burnout for *p* ≤ 0.2 were selected (Step 1). Then, stress was inserted (Step 2), followed by resilience (Step 3a) and satisfaction with life (Step 3b). In Step 4, the analysis was run, with all the variables of the previous steps included. 

### 3.1. The Mediating Effect of Resilience and Life Satisfaction on the Relationship between Stress and Exhaustion Burnout

**Figure 1:**
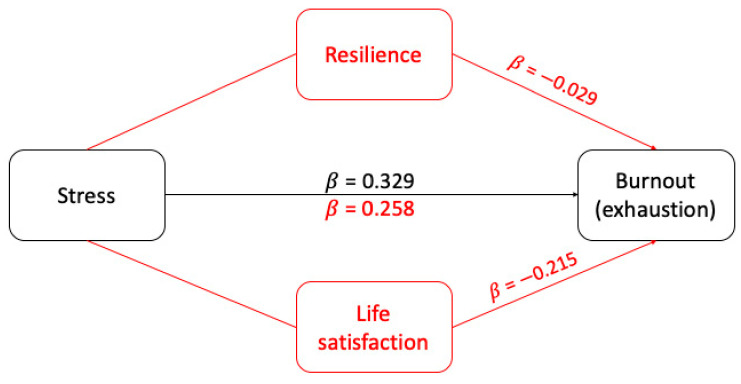
Diagram representing the modulating role of resilience and life satisfaction on the relationship between stress and exhaustion burnout. Changes in the value of beta when the modulator is present are noted in red.

The mediating role of resilience and life satisfaction on the relationship between stress and exhaustion burnout (outcome) was ascertained by means of linear regression analyses (Table 2). The selected variables were age, gender, civil status, have children, academic year, special status, leaves of the house how often, means, use of supplements and stimulants, physical exercise, extracurricular activities, stressful life events, psychological support, has taken a COVID test. The model obtained (Figure 1) demonstrated a positive association between exhaustion burnout and stress (β = 0.329, *p* < 0.001). In addition, there was a decrease in the association between stress and exhaustion burnout when analyzed with resilience and life satisfaction (β = 0.258, *p* < 0.001), with a negative association between resilience (β = 0.029, *p* = 0.001) and exhaustion burnout and between life satisfaction (β = −0.215, *p* = 0.002) and exhaustion burnout.

### 3.2. Mediating Effect of Resilience and Life Satisfaction on the Relationship between Stress and Disengagement Burnout

**Figure 2:**
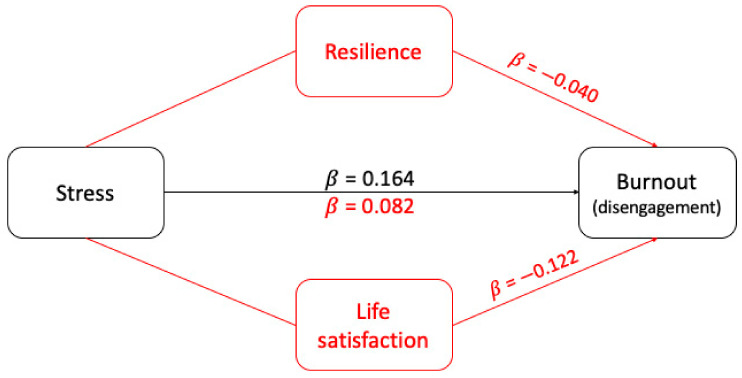
Diagram representing the modulating role of resilience and life satisfaction on the relationship between stress and disengagement burnout. Changes in the value of beta when the modulator is present are noted in red.

The mediating role of resilience and life satisfaction on the relationship between stress and disengagement burnout (outcome) was ascertained by means of linear regression analyses (Table 3). The selected variables included age, academic year, special status, leaving the house how often, volunteering, means, use of supplements, physical exercise, stressful life events, and psychological support. The model obtained (Figure 2) showed a positive association between disengagement burnout and stress (β = 0.164, *p* < 0.001). Similar to the previous case, there was a decrease in the association between stress and disengagement burnout when analyzed with resilience and life satisfaction (β = 0.082, *p* < 0.001) and a negative association between resilience (β = −0.04, *p* < 0.001) and life satisfaction (β = −0.122, *p* = 0.001) and disengagement burnout.

## 4. Discussion

The goal of the present study was to analyze the potential mediating roles of resilience and satisfaction with life on the relationship between perceived stress and burnout among medical students in the context of COVID-19. Indeed, in addition to the academic workload and responsibilities students bear as future health professionals with minimal margin for error, the epidemiological environment became another stress factor in undergraduate medical training [22,23]. For this reason, resilience and life satisfaction may have an impact on symptoms of anxiety and the subjective perception of well-being and, primarily, can function as protective variables against situations of burnout [40,41]. This study, therefore, presented the hypothesis that resilience and life satisfaction could mediate the association between perceived stress and burnout. In this case, two dimensions were considered: “disengagement burnout”, referring to burnout that manifests as a negative, cynical, and disconnected response to others; and “exhaustion burnout”, referring to the loss of emotional resources, resulting in mental and physical fatigue, lack of energy to complete tasks, and a sense of inability to restore one’s energy [5,59].

In analyzing the results, it was found that the value of the standardized regression coefficient that relates stress with “disengagement burnout” decreased after including resilience in the model and when adjusted for life satisfaction. This demonstrates that resilience and life satisfaction play a mediating role in the association between stress and “disengagement burnout”, with resilience exerting greater influence. These results are in alignment with previous studies indicating that resilience plays a role in managing emotions [59]. Considering the intrinsic characteristics of the medical profession and the demands inherent to caring for patients, it must be noted that this disengagement will have repercussions on the humane treatment and support of the patient. Thus, as expected, resilience and life satisfaction are negatively and significantly associated with stress and “disengagement burnout”.

As regards the coefficient value of the standardized regression relating stress with “exhaustion burnout”, a decrease was observed after including resilience in the model and when adjusted for life satisfaction. Therefore, it is once again underscored that resilience and life satisfaction negatively influence the association between stress and “exhaustion burnout”, with resilience exerting greater influence. These data reinforce what is described in the literature [40,41]. 

Previous models demonstrated that resilience and life satisfaction exert an influence on the association between stress and the various dimensions of burnout. However, other variables could interfere, namely the quality of social interactions, self-compassion, and a sense of purpose [60].

This study demonstrates, therefore, the need for institutions of higher education to develop policies of health promotion and self-care [4], which could include, for example, offering course subjects aimed toward developing stress management skills and building resilience [60]. Generally, mental health is associated with a considerable stigma, and in the case of medical students, there are several misconceptions about their mental health. Therefore, aiming to change such social perception regarding mental illness and the awareness of the need to look for treatment, the creation of groups for debate and dialogue within higher education institutions is highly recommendable. To develop interventions for the prevention or treatment of mental disorders and promote mental health, it is important to understand resilience, in particular the benefits of resilience training, which is particularly relevant for those professional groups who are more subject to a wide range of stress conditions. Another example might be offering psychoeducational mindfulness programs to increase psychological flexibility and resilience or taking action to raise awareness among this group in particular of the need to implement self-care strategies and the effects these can have on their role as caretakers. These measures are even more important for preventing burnout in situations like the COVID-19 pandemic. Effectively, the psychological, social, and economic impact of the epidemiological context makes it an additional stress factor, for which resilience is a tool that can help prevent burnout among medical students. All the findings of this study have contributed toward the understanding of the relationship between resilience, life satisfaction, stress, and burnout and have established that resilience and life satisfaction appear to be important factors in fighting burnout among future doctors. To the best of our knowledge, this study is of the first focus on mediating the role of resilience and life satisfaction in the relationship between stress and burnout in medical students.

Finally, our study has some limitations. First, it is based on an online survey shared through email and social networks and may therefore have been affected by a selection bias. In other words, students who have easier digital access or who are more aware of the topic may have been more likely to respond. Having used a convenience sample, our results may not be generalized to other populations and contexts. Furthermore, this was a transversal study, so the temporal interpretation of the data must be done with care. In this sense, given that these data refer to a specific and early period in the pandemic, it is also important to conduct a longitudinal study evaluating these effects in the long term, as well as to broaden the study to other Portuguese medical schools. Finally, it is suggested that there may be other variables affecting the relationship between stress and burnout; therefore, it could be useful to evaluate these variables in future studies. 

## 5. Conclusions

Burnout is a syndrome resulting from dimensions like exhaustion, which emerges as a consequence of intense emotional or physical pressure, in any occupational context, and disengagement, which involves distancing from and a lack of identification with the surrounding environment. With resilience being defined as the ability to maintain a state of equilibrium in response to stress, whereby goals are achieved with a minimum of psychological and physical distress, it has become pertinent to evaluate the way resilience and life satisfaction may influence and mediate the association between perceived stress and burnout among medical students in the context of the COVID-19 pandemic. 

This study has shown that when resilience and life satisfaction are analyzed in the association between stress and “disengagement burnout” and between stress and “exhaustion burnout”, there is a decrease in the relationship between stress and burnout. Despite these two variables having had a greater impact on the reduction related to “disengagement burnout”, the reduction in the two associations suggests that, despite stress and burnout having a clear positive association, the association can be minimized through mechanisms that promote resilience and greater life satisfaction.

Therefore, knowing that medical students are an emotionally vulnerable population with greater levels of stress and anxiety, and that resilience is associated with less burnout and greater tolerance for uncertainty, our study complements other existing studies in demonstrating that, in the current pandemic context, which has greatly compromised mental health, the promotion of institutional strategies to help develop mechanisms of adaptation to adverse events, as well as strategies to support students’ physical and psychological well-being based on resilience, improved perception of life satisfaction, and self-care, are fundamental.

## Figures and Tables

**Table 1:** Medical student characteristics (*n* = 462).

	*n* (%)
Sex	
Female	349 (75)
Male	113 (25)
Civil status	
Married/De facto union	10 (2)
Divorced/Single	452 (98)
Has children	9 (2)
Academic year	
1st	100 (22)
2nd	52 (11)
3rd	68 (15)
4th	67 (14)
5th	92 (20)
6th	83 (18)
Attending this academic year for the 1st time	443 (96)
Holds special status at faculty	60 (13)
Holds worker-student status	37 (8)
Is living away from home	87 (19)
Currently living with how many other people	
0	15 (3)
1	44 (10)
2	124 (27)
3	189 (41)
4 or more	90 (19)
Physical exercise	316 (68)
Leaves the house how often	
Occasionally	249 (54)
1–2 times per week	134 (29)
≥3 times per week	79 (17)
Has means for keeping up with classes	
Supplements/stimulants	
Extracurricular activities	197 (43)
Stressful life event	203 (44)
Psychological support	84 (18)
Has taken a COVID-19 test	
No, but would like to	154 (33)
No, doesn’t want to	298 (68)
Yes, has the result	10 (2)

**Table 2:** Results of multiple linear regressions (outcome: burnout exhaustion).

	Step 1 (β)	Step 2 (β)	Step 3a (β)	Step 3b (β)	Step 4 (β)
Male gender	−1.289	−0.226	−0.337	−0.407	−0.443
Age	0015	0.078	0.086	0.05	0.063
Civil status					
Married or non-marital partnership	Reference	Reference	Reference	Reference	Reference
Divorced or single	3.37	1.884	2.084	1.748	1.937
Has children	2.23	−0.819	−0.488	−0.578	−0.391
Academic year	−0.198	−0.131	−0.164	−0.508	−0.175
Special status	−0.852	−0.476	−0.493	−0.608	−0.585
Leaves the house how often					
Occasionally	Reference	Reference	Reference	Reference	Reference
1–2 times per week	−0.452	−0.375	−0.389	−0.258	−0.3
≥3 times per week	−0.678	−0.385	−0.337	−0.358	−0.329
Means for keeping up with classes	−2.107	−0.577	−0.496	−0.753	−0.644
Supplements/stimulants	0.991	0.317	0.389	0.371	0.411
Physical exercise	−1.578	−1.099	−0.887	−1.08	−0.924
Extracurricular activities	−0.467	−0.391	−0.227	−0.197	−0.124
Stressful life event	1.121	0.197	−0.206	0.124	0.15
Psychological support	1.674	0.776	0.527	0.575	0.439
Has taken a COVID test					
No, but would like to	Reference	Reference	Reference	Reference	Reference
No, doesn’t want to	−0.664	−0.515	0.466	−0.614	−0.049
Yes, has the result	0.757	−0.09	−0.158	−0.158	−0.55
Stress					
Resilience		0.329	0.276		0.258
Life satisfaction			−0.039		−0.029
				−0.17	−0.215

**Table 3:** Multiple linear regression results (outcome: burnout distancing).

	Step 1 (β)	Step 2 (β)	Step 3a (β)	Step 3b (β)	Step 4 (β)
Age	−0.02	0.012	0.023	−0.012	0.006
Academic year	−0.203	−0.097	−0.139	−0.139	−0.159
Special status	−0.887	−0.707	−0.69	−0.793	−0.749
Leaves the house how often					
Occasionally	Reference	Reference	Reference	Reference	Reference
1–2 times per week	0.077	0.132	0.113	0.257	0.198
≥3 times per week	−0.517	−0.319	−0.253	−0.296	−0.251
Volunteering	−0.606	−0.45	−0.469	−0.356	−0.404
Means for keeping up with classes	−1.085	−0.339	−0.198	−0.513	−0.338
Supplements/stimulants	−0.535	0.227	0.292	0.244	0.29
Physical exercise	−0.538	−0.291	0.001	−0.255	−0.031
Stressful life event	0.921	0.447	0.463	0.369	0.409
Psychological support	0.486	−0.03	−0.319	−0.219	−0.388
Stress		0.164	0.097	0.121	0.082
Resilience			−0.05		−0.04
Life satisfaction				−0.187	−0.122

## Data Availability

The exact data can be obtained from the corresponding author.
